# Microbiota and Drug Response in Inflammatory Bowel Disease

**DOI:** 10.3390/pathogens10020211

**Published:** 2021-02-16

**Authors:** Martina Franzin, Katja Stefančič, Marianna Lucafò, Giuliana Decorti, Gabriele Stocco

**Affiliations:** 1 Department of Medicine, Surgery and Health Sciences, University of Trieste, 34127 Trieste, Italy; martina.franzin@phd.units.it; 2 Department of Life Sciences, University of Trieste, 34127 Trieste, Italy; katja.stefancic@studenti.units.it (K.S.); stoccog@units.it (G.S.); 3 Institute for Maternal and Child Health—IRCCS “Burlo Garofolo”, 34137 Trieste, Italy; marianna.lucafo@burlo.trieste.it

**Keywords:** microbiota, microbiome, inflammatory bowel disease, pharmacotherapy

## Abstract

A mutualistic relationship between the composition, function and activity of the gut microbiota (GM) and the host exists, and the alteration of GM, sometimes referred as dysbiosis, is involved in various immune-mediated diseases, including inflammatory bowel disease (IBD). Accumulating evidence suggests that the GM is able to influence the efficacy of the pharmacological therapy of IBD and to predict whether individuals will respond to treatment. Additionally, the drugs used to treat IBD can modualate the microbial composition. The review aims to investigate the impact of the GM on the pharmacological therapy of IBD and vice versa. The GM resulted in an increase or decrease in therapeutic responses to treatment, but also to biotransform drugs to toxic metabolites. In particular, the baseline GM composition can help to predict if patients will respond to the IBD treatment with biologic drugs. On the other hand, drugs can affect the GM by incrementing or reducing its diversity and richness. Therefore, the relationship between the GM and drugs used in the treatment of IBD can be either beneficial or disadvantageous.

## 1. Introduction

Inflammatory bowel disease (IBD) refers to conditions characterized by chronic inflammation of the gastrointestinal (GI) tract and, in particular, comprises two main disorders, Crohn’s disease (CD) and ulcerative colitis (UC). CD and UC are distinguished by type of inflammation localization in the GI tract and symptoms but share some common features such as unknown etiology and extraintestinal manifestations [1]. 

Although the etiology of IBD still remains unknown, it is supposed to be caused by a combination of genetic susceptibility, environmental factors, microbial factors and an excessive immune response of the intestinal immune system, which damages the gut and causes symptoms [2]. As regards microorganisms, not only pathogens but also commensal bacteria are involved in the etiology of IBD because this disease leads to a loss of tolerance against the gut microbiota (GM) and, consequently, increases immunological reactivity against bacterial antigens; moreover, in IBD patients, there is an increased bacterial penetration of the intestinal mucosa and stimulation of the epithelium with the subsequent activation of immune cells, which promote the production of cytokines [3,4]. A disruption of the intestinal barrier or, more precisely, an increased intestinal permeability and mucus defects, can be a cause of IBD. A leakier intestinal barrier due mainly to a defective mucus because of an altered mucin production and composition leads to a higher antigen penetration, which causes an excessive stimulation of the intestinal immune system [5,6]. 

To date, a curative pharmacological therapy for IBD does not exist, and the treatment is aimed at improving symptoms, achieving mucosal healing, inducing and maintaining a steroid-free remission of active disease and avoiding relapses, hospitalization and surgery in order to improve the patient’s quality of life [7]. The main drugs used for the treatment of IBD are 5-aminosalicylic acid, corticosteroids, thiopurines, methotrexate and biologic drugs [7,8]. Biologic drugs used in IBD treatment include monoclonal antibodies that target and antagonize specific cytokines such as TNF-α, IL-12/IL-23 or prevent lymphocyte migration to the intestine by blocking α4-integrin or α4β7-integrin [9]. Antibiotics are also used in IBD, however their role in IBD pharmacotherapy is still not entirely clear, so they are mostly used to treat IBD complications [7]. Corticosteroids can be used to induce remission but not to maintain it because of their serious adverse effects during prolonged therapy, while thiopurines are mainly used to maintain remission because of their slow onset of action [7]. 

## 2. Association between the GM and IBD

The GM is strongly associated with the pathogenesis of IBD. In healthy individuals, the intestinal immune system is tolerant towards dietary antigens and the GM, and it is activated in presence of pathogenic organisms [10,11]. Instead, in IBD, the balance between the commensal microflora and the intestinal immune system is lost; either an excessive response of the immune system against non-pathogenic microbial organisms, the so-called loss of tolerance, is developed or there is no immune response towards an altered GM [10,12]. Alterations in GM, leading to a reduced diversity of the bacterial species, are sometimes referred as dysbiosis, and are associated with defective microbial functions provoking atypical immune responses and the development of various diseases [12,13]. Alterations in the intestinal immune system can contribute to the evolution of an abnormal microflora [14], but also the reshaping in the microbial community due to diet and pharmacological treatment can lead to aberrant immune responses by provoking an increased invasion and growth of pathogens [11,15,16]. Not only GM composition but also its function, including the production of bacterial metabolites, are altered during IBD. In particular, the proliferation of some species at the expense of others can result in a different profile of microbial metabolites such as short-chain fatty acids (SCFAs) and tryptophane [17,18].

Some bacterial species belonging to the GM may be involved in the protection against IBD. Indeed, sterile mice reconstituted with the commensal *Bacteroides thetaiotaomicron* (Bacteroidetes) present enhanced expression of genes with intestinal barrier functions and do not have an increment in the expression of proinflammatory genes [8,19]. Moreover, bacterial products such as SCFAs also have a protective role in IBD: butyrate has an anti-inflammatory effect because it increases the levels of Treg cells and reduces the production and activation of proinflammatory mediators and of T helper cells [17]. 

### Role of the GM in the Pathogenesis of IBD 

To date, there are three possible theories which consider the involvement of the GM in the pathogenesis of IBD: the development of IBD could be linked to (1) GM dysbiosis with an increase in pathogenic species and a parallel decrease in commensal bacteria, (2) altered immune responses to commensal bacteria, mainly caused by host genetic mutations and/or (3) an excessive translocation of bacteria because of a defective mucus barrier, which increases permeability [20]. 

There is evidence that supports the role of the microbiota in the development of IBD and other diseases: for instance, germ-free animals frequently do not develop intestinal inflammation, but inflammation occurs after colonization of their GI tract with some commensal bacteria [21,22]. Likewise, colonization of the gut of animal models by the microbiota derived from IBD donors can exacerbate colitis [23]. In the terminal ileum, in the colon, where bacteria are more abundant, and in the rectum, where there is stasis of fecal material, disease activity is at its highest. Furthermore, in IBD patients the mucosal bacterial population is more abundant compared to healthy individuals, and there are some polymorphisms in host genes linked to recognition and elimination of bacteria [23,24]. In addition, pharmacological treatment providing a long-term remission normalizes the microbiota in a majority of IBD patients [25]. 

The first theory associating the GM and IBD is linked to the presence of intestinal dysbiosis. Indeed, IBD patients have a GM depleted of bacteria with anti-inflammatory activities such as *Faecalibacterium prausnitzii* (Firmicutes) and enriched of bacteria with inflammatory functions that are able to adhere and invade the mucus layer [25,26]. More precisely, some bacteria belonging to the Firmicutes phylum (*Blautia faecis, Clostridium leptum, Clostridium lavalense, Faecalibacterium prausnitzii, Roseburia inulinivorans, Clostridiales, Lachnospiraceae*) are less abundant in IBD patients; instead, Bacteroidetes (*Bacteroides, Prevotella*), Actinobacteria, Proteobacteria (*Enterobacteriaceae,* in particular *Escherichia coli, Klebsiella pneumoniae, Pasteurellaceae*) and Fusobacteria (*Fusobacteriaceae*) are more abundant; reductions in the levels of Bacteroidetes and Proteobacteria and increments in some Firmicutes families have also been reported [25,27,28]. The microbial composition is also different between inflamed and non-inflamed intestinal areas: the inflamed sites of IBD patients are richer in species belonging to the *Prevotella* genus (Bacteroidetes) compared to non-inflamed sites [24].

Considering the dissimilarities between the two diseases, UC patients have lower levels of Firmicutes such as *Clostridium coccoides* and *Lactobacilli, Bifidobacteria* (Actinobacteria) [29] and *Enterobacteriaceae* (Proteobacteria) [30]. Additionally, in UC, invasive Fusobacteria are abundant, and there are higher levels of some bacterial populations belonging to the Proteobacteria phylum, such as *γ-Proteobacteria*, sulfate-reducing *δ-Proteobacteria* [30], the Bacteroidetes phylum (*Bacteroides fragilis,* especially enterotoxigenic *Bacteroides fragilis* (ETBF) and *Bacteroides vulgatus*) [31] and the Firmicutes phylum, such as *Ruminococcus gnavus* [32]. Compared to CD, UC has a higher percentage of bacteria, which translocate across the intestinal epithelial layers [31]. On the other hand, CD is characterized by an increase in some bacterial species belonging to the Fusobacteria phylum [31] and others belonging to the Proteobacteria phylum such as *Escherichia coli* [32]. Furthermore, in CD, there is a decrease in *Faecalibacterium prausnitzii* and *Clostridium leptum* (Firmicutes) [29,32]. In UC pediatric patients, there is an increase in *Bacteroides* (Bacteroidetes) and a decrease in *Lachnospiraceae* and *Lactobacillus* (Firmicutes); instead, in CD pediatric patients, the increased bacterial populations are *Enterobacteriaceae, Neisseriaceae, Pasteurellaceae* (Proteobacteria) and *Fusobacteriaceae* (Fusobacteria), and the decreased are *Bacteroidales* (Bacteroidetes) and some *Clostridiales* such as *Faecalibacterium prausnitzii,* but not *Veillonellaceae* (Firmicutes) and *Bifidobacteriaceae* (Actinobacteria) [33,34]. 

There are several mechanisms of action by which some microorganisms considered to be commensals can potentially become harmful: the so called pathobionts [35]. These microbes overgrow and can promote different diseases such as IBD only in determinate circumstances such as altered genetic or environmental conditions (modified GM composition or defective intestinal immune system) [36]. In other words, dysbiosis or genetic predisposition can lead commensal bacteria to shift from harmless to pathogenic [36,37,38]. 

Unlike pathobionts, beneficial bacterial levels are reduced in IBD. Both *Bacteroides* (Bacteroidetes) and *Clostridium* (Firmicutes), which have a role in reducing gut inflammation and increasing the number of Treg cells, are depleted in IBD. *Bacteroides fragilis* has an anti-inflammatory role because it regulates the balance between Th cells and Treg cells by secreting polysaccharide A (PSA); PSA also induces the production of interleukin (IL)-10. *Faecalibacterium prausnitzii (Clostridiales)*, which induces the increase in IL-10 and a decrease in proinflammatory cytokines such as IL-12, IL-17 and IFN-γ, is under-represented in IBD, especially in CD patients. Additionally, this bacterial species is involved in the development of Treg cells; it also abolishes the activation of nuclear factor kappa-light-chain-enhancer of activated B cells (NF-κB) and produces butyrate. *Faecalibacterium*, along with *Eubacterium rectale, Roseburia* (*Clostridiales*) and *Bacteroides thetaiotaomicron* (*Bacteroidales*), are four SCFAs-producing bacterial populations produced to a lesser extent in IBD. SCFAs such as butyrate are considered an energy source for enterocytes, have anti-inflammatory activities and reduce bacterial translocation by increasing the differentiation of goblet cells, thus leading to a higher production of mucus and, by regulating the role of claudins, the components of the tight junctions. Therefore, their deficiency in CD leads to barrier defects [32,38,39,40]. 

Another possible cause of gut dysbiosis may be explained by the oxygen hypothesis. The intestine of healthy individuals is mainly populated with obligate anaerobes as it contains low levels of oxygen due to oxygen consumption by facultative anaerobes immediately after birth [41]. In IBD, oxygen levels increase and disrupt the normal condition of anaerobiosis: aerobic and facultatively anaerobic species have an advantage, contrarily to obligately anaerobic organisms, which are disadvantaged, especially those who have the highest sensitivity to the presence of oxygen, such as *Faecalibacterium prausnitzii* (Firmicutes). The reasons why IBD is characterized by increased oxygen concentrations are the increased blood flow in the intestinal environment due to chronic inflammation. Gut inflammation generates both reactive oxygen species (ROS) and reactive nitrogen species (RNS), which provide oxidized products able to act as terminal respiratory electron acceptors in the anaerobic respiration [41,42]. As a consequence, it has been noticed that in IBD there is a reduction in obligately anaerobic bacteria (class *Clostridia* belonging to the Firmicutes phylum and class *Bacteroidia* belonging to the Bacteroidetes phylum) and a parallel increase in facultatively anaerobic bacteria (family *Enterobacteriaceae* belonging to the Proteobacteria phylum) [35,42]. 

As already mentioned, the loss of tolerance can be considered another link between IBD pathogenesis and the GM. The microbial dysbiosis present in IBD is regulated by the host genotype; the consequence is that some bacterial strains may be beneficial for one genotype and pathogenic for another [27]. In other words, shifts in bacterial composition are also caused by the predisposition due to genetic alterations, such as the ones relative to the *nucleotide binding oligomerization domain containing 2 (NOD2)* gene that induce modifications in bacterial sensing due to a lower activation of NF-κB in response to bacterial muramyl dipeptide or peptidoglycan [13,43]. For example, *Escherichia coli* (Proteobacteria) and *Enterococcus faecalis* (Firmicutes) initiate IBD only in genetically susceptible individuals; more precisely, both bacterial species are able to induce colitis if inoculated in gnotobiotic IL-10-deficient mice [44]. Moreover, variant alleles in both *NOD2* and *autophagy related 16 like 1* (*ATG16L1)* genes, which are associated with defects in autophagy and altered Paneth cells functions such as the secretion of antimicrobial peptides (AMPs), contribute to the reduction in *Faecalibacterium* (Firmicutes) and the increase in *Escherichia* (Proteobacteria). Indeed, AMPs, expressed dependently or independently of commensal bacteria, are thought to shape the composition of the GM [40,45,46,47]. 

Thirdly, an excessive bacterial invasion due to a defective mucus barrier also represents a link between the development of IBD and the GM. Indeed, in both UC and CD, there is an increase in the number of bacteria attached to the mucus layer [48]. *Ruminococcus gnavus* and *Ruminococcus torques* (Firmicutes) are mucolytic bacteria, which degrade the mucus layer to utilize its mucin glycans as an energy source. In homeostatic conditions, this mechanism is essential for ensuring a correct turnover of mucin proteins; however, if present in excessive concentrations, these bacterial species provoke an aberrant mucus degradation leading to an altered barrier permeability and an elevated invasion of bacteria, typical of IBD [32]. Intestinal barrier function is linked also to *Akkermansia muciniphila* (Verrucomicrobia)*,* a bacterial genus, which enhances barrier functions and has anti-inflammatory effects, and a decrease in *Akkermansia muciniphila* is found in IBD [32]. Additionally, in mice models associated with obesity and metabolic syndrome, this bacterium resulted to improve the functions of the intestinal barrier [49]. Other mucosal-adherent bacterial species abundant in IBD are *Enterococcus faecalis, Enterococcus faecium* (Firmicutes) and ETBF, enterotoxigenic *Bacteroides fragilis* (Bacteroidetes). The main bacterial populations that are able to transfer across the gut epithelium are *Escherichia coli* (Proteobacteria)*, Enterococcus faecalis, Staphylococcus* (Firmicutes)*, Bacteroides vulgatus* (Bacteroidetes) and *Fusobacterium varium* (Fusobacteria) [31].

Besides bacterial composition alterations, dysbiosis of the eukaryotic fungal GM (mycobiome) and of the virus GM (virobiota) have also been implicated in IBD pathogenesis. Indeed, some fungi and viruses are more abundant in IBD patients compared to healthy individuals; instead, others are decreased in both CD and UC. Fungi components may penetrate through the mucosal barrier by disrupting it, interact with receptors such as some lectin receptors, Toll-like receptors and members of the scavenger receptor family and, consequently, trigger immune responses. Viruses such as bacteriophages are also able to invade the gut epithelium and lead to immunological responses; additionally, they can change the bacterial ability to replicate [50,51]. 

## 3. The Relationship between Drugs and the GM

The GM has the ability to influence pharmacological effects, efficacy and toxicity of various drugs; however, these effects are still quite unknown because of the complexity of the host-GM relationship. The GM may act both on drugs’ pharmacodynamic and pharmacokinetic mechanisms, as it activates or inactivates medications and modulates their oral bioavailability or half-life through the production of microbial metabolites or enzymes, which interfere with drug metabolism and lead to active, inactive or toxic drug metabolites. The inter-individual variability in the GM composition may play a role in the development of individual responses to the same pharmacological therapy [52]. The contribution of the GM on various drugs has been reviewed elsewhere and in depth [53,54]. 

All drugs not completely absorbed in the upper intestine because of their low solubility or low permeability, drugs released exclusively in the colon or those administered rectally are capable of reaching the colon, where there is the greatest microbial density, allowing the interaction between the GM and the xenobiotics. Drugs administered intravenously or entirely absorbed in the upper GI tract can also arrive to the lower gut through mechanisms of secretion, diffusion from the blood or excretion from the bile [54].

Understanding how the GM affects the drug metabolism may help in predicting the therapeutic and toxicological actions of xenobiotics based on intra- and inter-individual variability, and to eventually obtain a personalized drug therapy [55]. 

There are many ways to study the formation of GM-derived metabolites, in particular by using intestinal contents or fecal suspensions derived from animals or humans or adding a xenobiotic to intestinal microbial enzyme sources. One of the most used techniques is liquid chromatography associated with mass spectrometry (LC/MS) [55]. It is often useful to associate the results obtained from the addition of a xenobiotic to gut microbial enzyme sources with in vivo animal studies, which identify the location of the metabolic activities as they compare bile and fecal metabolites. For instance, some metabolites derived from the antipsychotic drug risperidone are found only in rat feces and not in bile, suggesting that these molecules are produced by gut bacteria [54]. Furthermore, it is also possible to use the in vitro technique to study bacterial or mammalian cell cultures by using static, semi-continuous or continuous culture systems or by simulating the human GM [54,55]. Another in vitro approach consists of a system called the simulator of the human intestinal microbial ecosystem (SHIME), which is a five-stage multi-chamber system simulating the GI tract. The chambers representing the small intestine are inoculated with a human diet suspension; instead, the chambers representing the colon are inoculated with fecal suspensions in order to study the composition and activity of the GM [56,57]. Among these approaches, the most complete, but also invasive, way to study the microbial metabolic functions on drugs is the in vivo technique. This approach either quantifies lower and upper gut metabolites in differently released or administered formulations or compares GM-related drug metabolism in conventional animals with germ-free or gnotobiotic animals, which are used to study the role of a specific microbial component in the metabolism of a xenobiotic (involvement of the GM in drug metabolism if the metabolites are found in conventional animals but not in germ-free or gnotobiotic animals) [54]. 

## 4. Association between the GM and Drugs Used for the Treatment of IBD

The GM is able to influence the pharmacological activity of drugs used to treat IBD, and the same drugs are in turn capable of impacting on GM composition [58]. As already mentioned, some antibiotics are used in IBD especially to treat disease complications; these agents more than all other drugs affect microbial composition, however their association with the GM will not be discussed here because this review is focused on the use of immunomodulators in IBD pharmacotherapy.

### 4.1. Aminosalicylates

Despite its effective role in inducing, maintaining remission and preventing relapses in mild to moderate UC, the role of 5-aminosalicylic acid (ASA) in CD is still uncertain with reports showing that it may be slightly effective in clinically improving the disease or may not be effective [59,60]. 5-ASA has anti-inflammatory and immunosuppressive properties, as it leads to a reduced production of proinflammatory cytokines, decrease in NF-κB activation, inhibition of the synthesis of prostaglandins and leukotrienes, production of oxidized metabolites, which act as radical scavengers, inhibition of T cell synthesis, activation and differentiation; however, its precise mechanism of action is still unknown [61]. It is believed that the drug exerts its anti-inflammatory effects through colonic peroxisome proliferator-activated receptors-γ (PPARs-γ) by activating them and allowing their binding to peroxisome-proliferator response elements, genes involved in inflammation [62]. 

**Figure 1:**
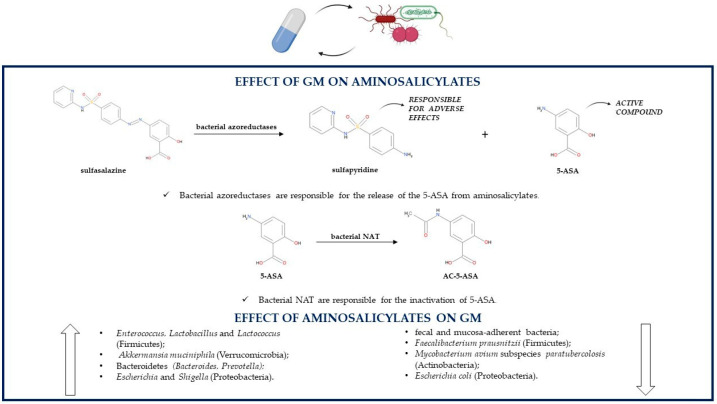
Association between the GM and aminosalicylates (5-ASA, 5-aminosalicylic acid; AC-5-ASA, 5-acetamidosalicylate; GM, gut microbiota; NAT, N-acetyltransferases).

Gut microorganisms produce azoreductases, enzymes able to reduce the azo bond of prodrugs such as sulfasalazine, so they are involved in the colonic release of the active, topically acting 5-ASA and systemically absorbed sulfapyridine (Figure 1); the latter is primarily responsible for the side effects of sulfasalazine such as nausea, anorexia and skin rush. After sulfasalazine treatment the feces of conventionally treated animals do not contain the prodrug; instead, antibiotic-treated or germ-free animal models present with unmodified sulfasalazine in their feces. Since CD, on the contrary of UC, is not always localized in the colon, it is clear that 5-ASA is more effective in UC, as its release from sulfasalazine occurs exclusively in the colon [54]. 

As already mentioned, sulfasalazine side effects are due mainly to sulfapyridine, so the medication can be replaced by another prodrug, olsalazine, which releases only 5-ASA. Indeed, olsalazine contains two 5-ASA molecules linked together by an azo bond. Similarly to sulfasalazine, olsalazine’s azo bond can also be cleaved by azoreductases produced by some bacterial species belonging to the GM. After olsalazine administration, its metabolite 5-ASA is found only in the urines of healthy individuals and not in those who previously underwent an ileostomy, confirming that, without the action of the GM, there is no olsalazine conversion to 5-ASA [54,63]. 

Sulfasalazine, olsalazine and balsalazide, which consists of 5-ASA linked through an azo bond to an inert molecule (4-aminobenzoyl-β-alanine), cannot be absorbed in the upper GI tract and so cannot be metabolized by the host to the active 5-ASA, which makes even more important the role of the GM in producing the enzymes needed for cleaving the azo bond of these prodrugs; indeed, colonic bacteria are the only ones able to release 5-ASA from its prodrugs. Interestingly, the three medications are characterized by inter-individual variability in therapeutic responses because of different rates of azo-bond cleavage. Sulfasalazine degradation is faster compared to the degradation of balsalazide and olsalazine, which indicates that azoreductases’ affinity and activity depend on structural features of their target molecules [64]. The main bacterial species producing azoreductases are anaerobes and belong to the genera *Clostridium* (*Clostridium clostridiiforme, Clostridium nexile, Clostridium paraputrificum, Clostridium perfrigens*) and *Eubacterium* (*Eubacterium hadrum*) of the Firmicutes phylum, but also *Staphylococcus aureus* (Firmicutes), *Escherichia coli, Klebsiella aerogenes,* some *Pseudomonadaceae* (Proteobacteria) and several species of *Bacteroides* (Bacteroidetes) can produce these enzymes [65]. 

Both 5-ASA and sulfapyridine can undergo an acetylation reaction leading to the inactive products 5-acetamidosalicylate (AC-5-ASA) and N-acetylsulfapyridine (AC-SP), respectively. Intestinal bacteria are responsible for the inactivation of 5-ASA and sulfapyridine, by producing AC-5-ASA more quickly than AC-SP. In animal models, the recovery of 5-ASA depends on the species, as it is greater in rats than in guinea pigs; this difference is due to a different ability of the GM from various species to induce the acetylation reaction [66]. 

The acetylation takes place in aerobic and anaerobic conditions, so it is carried out by both aerobic and anaerobic gut bacteria [67]. N-acetyltransferases (NAT) are the enzymes responsible for 5-ASA and sulfapyridine acetylation, and bacterial organisms able to produce NAT are Proteobacteria, especially genera *Citrobacter, Escherichia* (*Escherichia coli*)*, Klebsiella* (*Klebsiella pneumoniae*), *Morganella, Plesiomonas, Pseudomonas* (*Pseudomonas aeruginosa*)*, Serratia, Shigella* and *Vibrio*, but also genera *Bacteroides* (Bacteroidetes)*, Clostridium* and *Lactobacillus* (Firmicutes). *Pseudomonas aeruginosa* is the strongest N-acetylator among the studied bacterial species. In addition, NAT isoforms are also present in humans, so they may contribute to the aminosalicylate acetylation [68]. Despite being influenced by the GM, 5-ASA can at the same time influence the microbial population of IBD patients; the association between 5-ASA and inflammation-related bacteria may be one of the causes of its treatment efficacy. Compared to untreated UC patients, 5-ASA treatment increases the levels of some Firmicutes genera such as *Enterococcus, Lactobacillus* and *Lactococcus* and reduces the levels of *Faecalibacterium prausnitzii* (Firmicutes)*, Akkermansia muciniphila* (Verrucomicrobia)*,* Bacteroidetes *(Bacteroides, Prevotella)* and Proteobacteria such as *Escherichia* and *Shigella*; higher concentrations of Firmicutes, especially those producing SCFAs, are negatively correlated with UC severity; instead, the abundance of pro-inflammatory Proteobacteria is positively associated with disease severity [69]. 5-ASA can also lead to a lower abundance of fecal and mucosa-adherent bacteria. There are some possible mechanisms by which 5-ASA influences the GM. First, a decrease in the gut luminal pH is observed, because of 5-ASA’s metabolite acetylsalicylic acid reduces the levels of *Akkermansia muciniphila* (Verrucomicrobia), a species resident in the distal colon that prefers a higher pH, and contributes to an increase in the beneficial *Bifidobacteria* (Actinobacteria) and *Lactobacilli* (Firmicutes). Additionally, this medication reduces the expression of proinflammatory cytokines and the destruction of tight junction proteins, thus enhancing the mucosal barrier functions and limiting bacterial translocation. However, lower concentrations of *Akkermansia muciniphila* (Verrucomicrobia), a mucin-regulator producing SCFAs and monosaccharides needed by some butyrate-producing microbial organisms for their growth, reduce mucosal barrier protection [70,71]. 5-ASA can inhibit the growth of some pathogens [72]. It is still not known if the infectious pathogen *Mycobacterium avium* subspecies *paratuberculosis* (MAP) (Actinobacteria) could be involved in the etiology of IBD. 5-ASA treatment reduces the growth of this species in a dose-dependent manner acting as an anti-MAP antibiotic. On the contrary, sulfapyridine does not have any effect on MAP growth. Interestingly, if considering *Mycobacterium avium* subspecies *avium* (Actinobacteria), 5-ASA does not influence its growth, but sulfapyridine is as effective as methotrexate, of which was hypothesized an anti-MAP action, against this pathogen [72]. 5-ASA also reduces the expression of IBD-related bacterial virulence genes and limits the growth of *Escherichia coli* (Proteobacteria) in a dose-dependent manner by inhibiting the transcription of *Escherichia coli* virulence genes related to IBD and to colorectal cancer; however, its mechanism of action needs further elucidation. As a consequence, 5-ASA reduces *Escherichia coli* infectivity by lowering its motility, adherence and invasion of gut epithelium, survival in macrophages, NF-κB activation and thus proinflammatory IL-8 secretion, TNF-α production, stress, DNA damage, and by enhancing PPAR-γ gene expression [73]. 5-ASA influences the growth of IBD-related *Campylobacter concisus* (Proteobacteria) by inhibiting the growth of some strains and by increasing the growth of other strains; however, the reason behind these differences is unclear. The inhibition of this bacteria may represent another therapeutic action of 5-ASA; instead, the promotion of its growth may be the cause of 5-ASA-related exacerbations of colitis [74]. One of the causes of 5-ASA’s therapeutic effect is the fact that it reduces IBD-related hydrogen sulfide production with an unknown mechanism. Toxic hydrogen sulfide is produced either from sulfate by gut sulfate-reducing bacteria or from amino acids firstly by amino acid-fermenting bacteria such as *Fusobacterium necrophorum* (Fusobacteria) and secondly by human thiol S-methyltransferase. The order of effectiveness of equimolar concentrations of 5-ASA drugs in reducing sulfide production is sulfasalazine, then olsalazine, balsalazide and lastly 5-ASA; instead, sulfapyridine does not influence hydrogen sulfide levels. Sulfasalazine is more effective than 5-ASA in inhibiting hydrogen sulfide production by influencing both amino acid-fermenting bacteria and sulfate-reducing bacteria [75] (Table 1). 

### 4.2. Corticosteroids

Corticosteroids such as cortisone, hydrocortisone, methylprednisolone, prednisolone, prednisone, beclometasone and budesonide are used to induce remission in both CD and UC [76]. First-generation corticosteroids such as methylprednisolone and prednisone are systemically used to induce remission; several second-generation drugs such as budesonide and beclomethasone dipropionate, which are used topically, have recently been introduced, because their adverse reactions are less frequent and less severe compared to first generation corticosteroids [77,78,79,80]. Corticosteroids exert their anti-inflammatory effects by binding to their glucocorticoid receptor (GR) in the cytosol of immune cells, which leads to conformational changes of the receptor and the translocation of the complex drug-receptor to the nucleus, where it binds to the DNA and regulates the expression of genes involved in inflammatory responses. In this way, steroids suppress the activation and differentiation of immune cells, for example T and B cells, reduce the production of proinflammatory proteins such as NF-κB and their derived cytokines such as tumor necrosis factor (TNF)-α, IL-1α, IL-1β, IL-6 and IL-8 and increase the production of anti-inflammatory cytokines including IL-10; additionally, steroids induce apoptosis of T cells and dendritic cells [77,79,81]. 

**Figure 2:**
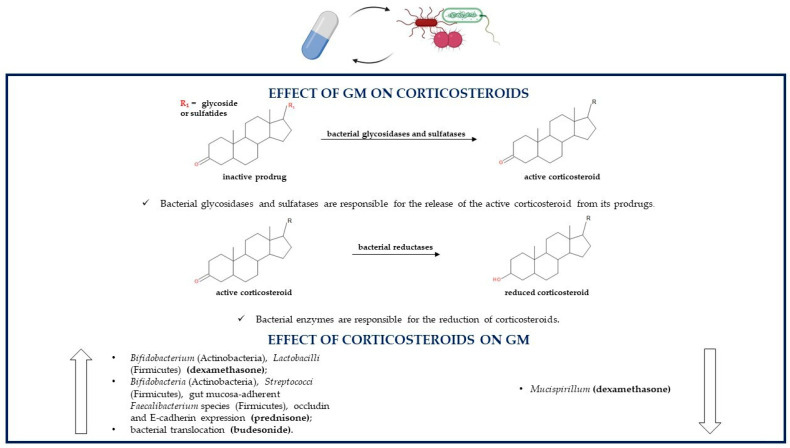
Association between the GM and corticosteroids (GM, gut microbiota).

GM is able to produce enzymes that can metabolize and degrade corticosteroids. Indeed, these drugs can be administered as prodrugs, which undergo GM-related hydrolysis by enzymes such as glycosidases and sulfatases or in pH-dependent formulations (Figure 2). 

Both techniques allow the release of the pharmacologically active steroid in the distal small bowel and proximal colon where they are directly in contact with the GM; because of enterohepatic circulation also parenterally administered glucocorticoids can undergo bacterial metabolism. Lastly, glucocorticoids used in IBD treatment can endure a GM-related reductive reaction. Metabolic susceptibility and stability of these drugs depend on their chemical structures; for example, beclometasone and its derivative beclometasone dipropionate are less influenced by GM-mediated gut metabolism compared to other steroids so they are less likely to be transformed by the GM [82,83]. Following incubation in a fecal inoculum to simulate the gut lumen, prednisolone is degraded within three hours, its prodrug prednisone within 96 h, budesonide within seven hours (with its S epimer being metabolized more rapidly compared to the R epimer maybe because of their different structural conformations) and beclometasone dipropionate within two hours; instead, beclometasone dipropionate’s active product beclometasone-17-monopropionate (17-BMP) remains quite stable. On the contrary, these corticosteroids are not metabolized in the absence of human feces, which confirms the role of the GM in their degradation [84]. As regards budesonide, the data are not univocal. Indeed, a study showed that bacterial degradation of budesonide in distal small intestine and proximal colon is not clinically relevant because the half-life of the xenobiotic was measured to be 203 and 147 min in simulated ileal bacteria and simulated colonic bacteria, respectively. The degradation half-life of budesonide was estimated by incubating the drug with fecal material simulating ileal bacteria to verify the drug degradation in the distal small intestine or simulating colonic bacteria to verify the drug degradation in the proximal colon [85]. Corticosteroids are also able to modulate the GM; indeed, some drugs belonging to the corticosteroid family regulate bacterial composition; instead, others have no influence on the GM. For instance, prednisolone shows no effect on colonic microflora of dogs, so its anti-inflammatory activity is not related to the GM modulation [86]. On the contrary, other corticosteroids such as dexamethasone increase the levels of *Bifidobacterium* (Actinobacteria) and *Lactobacilli* (Firmicutes) and decrease the levels of *Mucispirillum*, a gut mucin degrader. Corticosteroid-mediated microbial regulation leads to the consequent downregulation of *Muc2* gene expression in proximal colonic goblet cells; Muc2 is the most important mucin component of the mucus layer. This alteration is absent in germ-free mice, indicating that corticosteroids alone are not enough to alter mucus production and gut barrier protection [87]. Prednisolone’s prodrug prednisone affects canine gut bacterial population by increasing the levels of *Bifidobacteria* (Actinobacteria) and *Streptococci* (Firmicutes) in all mucosal areas and by enhancing the percentage of gut mucosa-adherent *Faecalibacterium* species (Firmicutes); these bacteria may all play a role in inducing remission in IBD. Prednisone therapy leads also to an increment in occludin and E-cadherin expression, which are important proteins in tight junctions and adherent junctions, respectively, that modulate epithelial adhesion and intestinal barrier functions [88]. Experimental data have shown that budesonide, and by extension, maybe also other glucocorticoids, regardless of the beneficial anti-inflammatory activity in IBD, can also provoke harmful effects. Indeed, they indirectly act on GM activities by enhancing bacterial translocation through the reduction in mucosal barrier functions and the decrease in the levels of claudin-2 and claudin-4, important components of the tight junctions. Bacterial translocation from the gut to extraintestinal tissues provokes an abundance of aerobic and anaerobic bacteria in mesenteric lymph nodes and in the liver. All these budesonide-related effects may lead to an increase in the risk of developing sepsis and in a dose-dependent deterioration of clinical conditions [89]. The mechanisms of the alterations of steroids such as dexamethasone, prednisone and budesonide on the microbial population are still not clear [87,88,89] (Table 1). 

### 4.3. Azathioprine and Mercaptopurine

Azathioprine (AZA) and mercaptopurine (MP) are two immunosuppressant drugs belonging to the thiopurine class. They are used only in the maintenance therapy of both CD and UC [60,90]. Thiopurines have a complex cellular pathway. Because of their chemical structure similar to purines, they exert their therapeutic activity in lymphocytes acting as antimetabolites. Both AZA and MP are prodrugs, and they are converted in thioguanine nucleotides (TGNs), which are incorporated into DNA and RNA of leucocytes, where they disrupt DNA and RNA replication leading to the arrest of the cell cycle and apoptosis. Thioguanine-triphosphate also suppresses GTPase Rac1 activation by acting as a substitute of GTP, therefore provoking apoptosis of T lymphocytes. Additionally, thiopurines can suppress the activity of phosphoribosyl pyrophosphate amidotransferase, the enzyme that catalyzes the first reaction in the de novo purine synthesis, leading to cytotoxicity [91,92,93].

**Figure 3:**
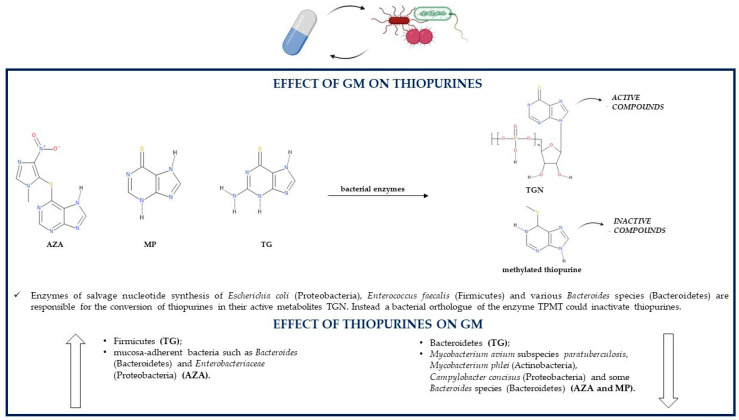
Association between the GM and thiopurines (AZA, azathioprine; GM, gut microbiota; MP, mercaptopurine; TG, thioguanine; TGN, thioguanine nucleotides; TPMT, thiopurine S-methyl transferase).

As shown in Figure 3, bacteria belonging to the GM can convert thiopurine drugs such as MP and TG in the active TGN. Indeed, it was noticed that the *Escherichia coli* (Proteobacteria) DH5α strain is able to metabolize both MP and TG with a consequent production of TGN that is threefold higher in case of TG. This difference may be due to different metabolic pathways of the two thiopurines: TG is directly metabolized to TGN as a result of hypoxanthine phosphoribosyl transferase (HPRT) enzyme activity; instead, MP conversion to its active metabolites is indirect as it requires a few steps, one of which, an inosine-5′-monophosphate dehydrogenase (IMPDH)-related enzymatic reaction, could limit the production of TGN [94]. The bacterial-mediated conversion of TG to active TGN is confirmed by the fact that TG can be metabolized into TGN even in the absence of the host enzyme HPRT, so the microbial metabolism of thiopurines is able to ameliorate colitis and reduce gut inflammation on his own. Bacterial species able to transform TG to TGN are *Escherichia coli* (Proteobacteria), as already mentioned, but also *Enterococcus faecalis* (Firmicutes) and *Bacteroides thetaiotaomicron* (Bacteroidetes) [95].

Some gut bacteria, especially those belonging to the Proteobacteria phylum, could induce methylation reactions because they synthesize an orthologue of the human thiopurine S-methyl transferase (TPMT) enzyme [96]. The bacterial orthologue of the human TPMT was found in *Pseudomonas syringae* (Proteobacteria). The enzyme belongs to the S-adenosylmethionine (SAM)-dependent methyltransferases, such as human TPMT, so it may be able to catalyze the methylation of thiopurines and, thus, simultaneously reduce their conversion to active TGN; however, this has not yet been proven [97]. TPMT activity was reported also in other bacterial species such as *Pseudomonas aeruginosa, fluorescens* and *ovalis* (Proteobacteria) [98].

A study about the effects of thiopurines and 5-ASA on the growth of five different bacterial species reported that some of these species are also able to produce enzymes involved in the thiopurine metabolic pathway; the production of such enzymes may have a role in influencing the effects of AZA and MP on the growth of these bacteria. Interestingly, in *Bacteroides fragilis* (Bacteroidetes) and *Enterococcus faecalis* (Firmicutes), some bacterial enzymes essential for the conversion of thiopurine drugs into active TGN are present, more precisely HGPRT (or HPRT), IMPD (or IMPDH) and guanosine monophosphate synthetase (GMPS). Additionally, *Bacteroides vulgatus* (Bacteroidetes) and *Escherichia coli* (Proteobacteria) contain all the necessary enzymes (HPRT, IMPD and GMPS) for the metabolic transformation of AZA to TGN; *Escherichia coli* (Proteobacteria) is able to produce the XO enzyme too, which leads to the synthesis of inactive thiopurine metabolites [74].

Despite being able to maintain remission in IBD patients by decreasing the levels of lymphocytes and proinflammatory mediators in the inflamed tissues, restoring goblet cell morphology, enhancing mucin expression and mucin production, inducing epithelial cell antibacterial autophagy and epithelial intracellular bacterial killing, thiopurines act by directly modulating the GM. Indeed, in mouse models, TG does not induce any alteration in luminal gut bacteria, but it influences the composition of mucosa-adherent bacteria by decreasing Bacteroidetes levels and increasing Firmicutes levels [95]. In IBD patients, MP treatment reduces GM richness and diversity, so it may be partially responsible for gut dysbiosis [96,99]. An in vitro study reported that both TG and MP interfere with DNA synthesis and functions in the toxic *Bacillus cereus* (Firmicutes), leading to damaged bacterial nucleic acids and reduced RNA and protein synthesis, which suggests that thiopurines may have antibacterial effects; however, the mechanism for these effects is not known [94]. 

AZA and MP have a dose-dependent bacteriostatic activity against *Mycobacterium avium* subspecies *paratuberculosis* (MAP) (Actinobacteria), one of the possible risk factors of IBD development. Therefore, the improvement in IBD clinical signs following AZA or MP therapy may be related to the treatment of a MAP infection through the inhibition of MAP growth, which may lead to another thiopurine therapeutic effect, the reduction in proinflammatory cytokines. The potency of the thiopurine effects on this bacterium is similar to the potency of the antibiotic ciprofloxacin, especially as regards MP. Both thiopurines are also capable of inhibiting the growth of *Mycobacterium phlei*; however, they do not act on all *Mycobacterium* species as *Mycobacterium avium* and *Mycobacterium smegmatis* are resistant to thiopurine antibacterial effects. Additionally, *Escherichia coli* (Proteobacteria) and *Enterococcus faecalis* (Firmicutes) are not influenced by thiopurine treatment, suggesting that the antibacterial effects of thiopurines might be specific for some bacterial species [100,101]. AZA and MP can affect the growth of other IBD-related species such as the Gram-negative *Campylobacter concisus* (Proteobacteria) and some other enteric bacteria, which suggests another supplementary therapeutic mechanism of these medications. More precisely, both thiopurine drugs inhibit in a significant manner the growth of some *Campylobacter concisus* strains, which are very sensitive especially to the presence of AZA. AZA is also more effective than MP in inhibiting the growth of some IBD-associated *Bacteroides* species such as *Bacteroides fragilis* and *Bacteroides vulgatus* (Bacteroidetes); interestingly, its imidazole ring is a typical structural feature of some antimicrobial drugs such as metronidazole. MP is more effective on *Bacteroides fragilis* than on *Bacteroides vulgatus*. Between AZA and MP, only the former is able to affect *Escherichia coli* (Proteobacteria) growth, but only at high drug concentrations [74].

AZA is able to significantly reduce the migration of leukocytes to inflamed tissues and to reduce leukocyte concentrations in the intestinal mucus, which still, however, remain higher compared to those of healthy individuals. This thiopurine increases mucosal adherence of bacteria and can enhance by a thousand-fold the levels of mucosa-adherent bacteria in comparison to healthy controls. For instance, following AZA treatment, there is a greater adhesion to the GM of bacteria such as *Bacteroides* (Bacteroidetes) and *Enterobacteriaceae* (Proteobacteria). The AZA-related increase in mucosal bacteria is caused by the fact that this drug leads to an increment in amenability, an indirect indication of bacterial vitality diminished in IBD probably because of leukocyte migration and inflammatory responses, both of which are reduced by AZA treatment [102]. In CD patients responding to AZA, the levels of Proteobacteria are significantly decreased and the levels of Bacteroidetes are increased; *Bacteroides* (Bacteroidetes) abundance is associated with remission. In individuals suffering from CD, especially in those in remission due to AZA therapy, there is an increment in microbial butyrate production. On the contrary, CD patients who cannot maintain remission with thiopurines have high levels of *Lactobacillus* (Firmicutes) and *Klebsiella* (Proteobacteria), which are both representative of a persistent disease [103] (Table 1).

### 4.4. Methotrexate

Methotrexate (MTX) is an antifolate drug, that is a methyl analogue of folic acid with antimetabolite functions [80,104]. MTX and its metabolites act by inhibiting various enzymes in the metabolic pathway of folic acid. More precisely, high doses of MTX are used in oncology because they have cytotoxic and antiproliferative effects due to the inhibition of dihydrofolate reductase and thymidylate synthase, leading to an inhibition of the purine and pyrimidine synthesis [105,106,107,108]. Instead, lower doses of the drug are used in immune-mediated diseases such as CD because of anti-inflammatory and immunomodulatory actions due to the suppression of other folate-dependent enzymes [109]. MTX and especially its metabolites polyglutamates are capable of inhibiting 5-aminoimidazole-4-carboxamide ribonucleotide (AICAR) transformylase; subsequently, there is an adenosine accumulation [107,110]. Adenosine exerts antioxidant and anti-inflammatory effects by both regulating the CD39/CD73 axis, involved in its synthesis, and binding to adenosine receptors [108,111,112]. 

The inhibition of dihydrofolate reductase, thymidylate synthase and AICAR transformylase depends on both MTX and its polyglutamates. Indeed, MTX in its mono-glutamate state, also referred to as MTX-PG1, is metabolized by the liver and by the gut to hydroxy-MTX (7-OH-MTX), which is transported to the GI tract. In the gut, both MTX-PG1 and 7-OH-MTX are transported into the cells where they are metabolized by folylpolyglutamate synthetase (FPGS) into MTX polyglutamates (MTX-PGs), which are not able to get out of cells because of their low affinity for folate transporters. MTX-PGs are subsequently reconverted by human γ-glutamyl hydrolase or bacterial glutamate carboxypeptidase II to MTX-PG1, which is again able to leave the cells [113,114,115].

**Figure 4:**
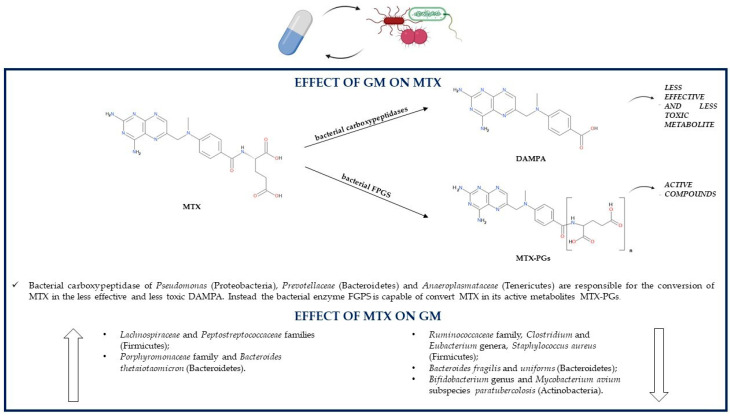
Association between the GM and methotrexate (DAMPA, 4-diamino-N-10-methylpteroic acid; GM, gut microbiota; FPGS, folylpolyglutamate synthetase; MTX, methotrexate; MTX-PGs, MTX polyglutamates).

The role of the GM in the metabolism of MTX via hydrolysis was firstly supposed when a study showed an increase in mortality of MTX-treated mice if they were previously treated with antibiotics, which may be related to the fact that the GM can convert MTX into a nontoxic metabolite; instead, a GM altered by antibiotic use cannot biotransform MTX. Another study supporting the association between the GM and MTX metabolism demonstrated that after radiolabeled MTX treatment, germ-free mice had a higher rate of radioactivity than controls, suggesting again that gut bacteria can degrade MTX [82]. Indeed, the major metabolite obtained from MTX GM conversion is 4-amino-4-deoxy-N10-methylpteroic acid (APA), and the enzyme responsible for the hydrolysis is supposed to be a bacterial carboxypeptidase. APA is a less effective inhibitor of dihydrofolate reductase than MTX but is also less toxic than its precursor [116]. The conversion of the mono-glutamate form of MTX to MTX-PGs can be performed by the patients’ folylpolyglutamate synthetase (FPGS) but also by some bacterial species [115] (Figure 4). 

The subsequent MTX-PGs reconversion to MTX-PG1 can be performed through the hydrolysis reaction of an enzyme produced by some *Pseudomonas* (Proteobacteria) strains, which utilize the glutamate derived from the reaction as a source of carbon and nitrogen [117]. Both mono-glutamate MTX and its metabolite 7-OH-MTX can be further metabolized by the gut bacterial enzyme glutamate carboxypeptidase II, in particular, by the *Escherichia coli* (Proteobacteria) orthologue enzyme p-aminobenzoylglutamate hydrolase to 2,4-diamino-N-10-methylpteroic acid (DAMPA) and 7-hydroxy-DAMPA, respectively. These two products are inactive, so they contribute to the detoxification of the organism from MTX, which is fundamental especially in patients with delayed drug clearance and/or MTX-related adverse effects. Despite *Pseudomonas* (Proteobacteria), other bacteria able to synthesize glutamate carboxypeptidase II might be *Prevotellaceae* (Bacteroidetes) and *Anaeroplasmataceae* (Tenericutes); indeed, studies showed that the presence of DAMPA in the feces is positively associated with these bacterial families [114].

Regardless of having an antiproliferative or immunosuppressive role, MTX is associated with some adverse reactions too; for instance, those of the gastrointestinal tract. The mechanisms by which MTX is able to induce gastrointestinal damage include alterations in the GM composition through the stimulation of the growth of M1 proinflammatory macrophages, which have a role in recognizing and eliminating bacteria, and through the reduction in the M2 anti-inflammatory macrophage phenotype. This results in a decreased total GM diversity; more precisely, in mice treated with MTX, there is a decrement in the levels of *Ruminococcaceae* (Firmicutes) and Bacteroidetes, especially as regards the order *Bacteroidales* and species *Bacteroides fragilis* and *Bacteroides uniforms*; instead, levels of *Lachnospiraceae* (Firmicutes) and *Bacteroides thetaiotaomicron* (Bacteroidetes) increase. *Bacteroides fragilis* (Bacteroidetes) is able to ameliorate MTX-related gastrointestinal toxicity, so its decrease aggravates inflammatory responses and macrophage alterations [118]. Another study in healthy male Sprague-Dawley rats showed that low-dose MTX treatment increases the concentrations of Firmicutes over those of Bacteroidetes; instead, the GM alterations are exactly the opposite in case of high-dose MTX administration. High doses of MTX increment *Peptostreptococcaceae* (Firmicutes) and *Porphyromonadaceae* (Bacteroidetes) levels and reduce *Clostridium, Eubacterium, Ruminococcus* (Firmicutes) and *Bifidobacterium* (Actinobacteria) levels; indeed, high concentrations of this drug can inhibit the growth of almost a half of the representative gut bacteria. Additionally, long-term exposure to MTX or the use of high doses have deleterious effects on the GM, as they induce some perturbations leading to an altered glutamate carboxypeptidase II activity, which reduces MTX conversion to the nontoxic DAMPA and thus increases MTX-related gastrointestinal toxicity [114]. MTX is able to inhibit the growth of the IBD-associated pathogenic microorganism *Mycobacterium avium* subspecies *paratuberculosis* (Actinobacteria), so its therapeutic actions may not only be related to the antiproliferative or anti-inflammatory effects, but also to the treatment of MAP infections; however, it is still unknown if the eradication of MAP could be beneficial in IBD [100]. In vitro, the pathogen *Staphylococcus aureus* (Firmicutes) can be inhibited by MTX, which has on this microorganism an inhibition efficacy comparable to that of commonly used antibiotics. On the contrary, this drug has no antibacterial activity against bacterial strains of *Escherichia coli* and *Pseudomonas aeruginosa* (Proteobacteria) and against the fungal strain *Candida albicans* [119] (Table 1).

### 4.5. Biologic Drugs

All biologic drug families used to treat IBD can change GM composition, and this may influence clinical responses to treatment. Indeed, TNF-α inhibitors, integrin receptor antagonists and interleukin antagonists are all able to indirectly increase microbial diversity. Common patterns of GM alterations due to treatment with biologics include an increment in the abundance of SCFAs-producing bacteria and a reduction in *Escherichia* (Proteobacteria) and *Enterococcus* (Firmicutes) [120].

#### 4.5.1. TNF-α Inhibitors

Anti TNF-α antibodies may be divided in first-generation inhibitors such as infliximab and adalimumab and second-generation biologicals such as certolizumab pegol, golimumab and infliximab biosimilars [121,122]. In mild and moderate IBD, TNF-α inhibitors represent an alternative to conventional therapy, so are beneficial in patients who are refractory or dependent of corticosteroids and/or immunosuppressive agents; on the other hand, in severe IBD, these drugs are a part of the conventional treatment [123,124]. Anti TNF-α agents act by inhibiting the proinflammatory action of the cytokine TNF-α, whose levels are increased in both gut mucosa and serum of IBD patients. TNF-α presents two forms: the transmembrane protein, with a role in inducing and maintaining inflammation, and the soluble form, the main form responsible for TNF-α effects such as tissue injury and stimulation of other immunomodulatory molecules, cell proliferation, differentiation and apoptosis, which leads to B cell activation, proinflammatory cytokine expression, higher intestinal permeability and the accumulation of neutrophils and adhesion molecules [122,123]. It is supposed that anti TNF-α antibodies act by two different mechanisms in IBD patients. Firstly, they are able to rapidly induce apoptosis of T cells. Indeed, these drugs can block the transmembrane TNF-α-induced antiapoptotic signaling or directly bind to the transmembrane form of the cytokine and consequently avoid the binding to its receptor, but it cannot be excluded that they also bind to the soluble form of TNF-α bound to its receptor present on T cells. Anti TNF-α antibodies may also prevent the processing of the transmembrane form of TNF-α to its soluble form. Secondly, TNF-α drugs containing an Fc region can induce M2-type wound-healing macrophages in responders to therapy and thus contribute to mucosal healing [125]. 

**Figure 5:**
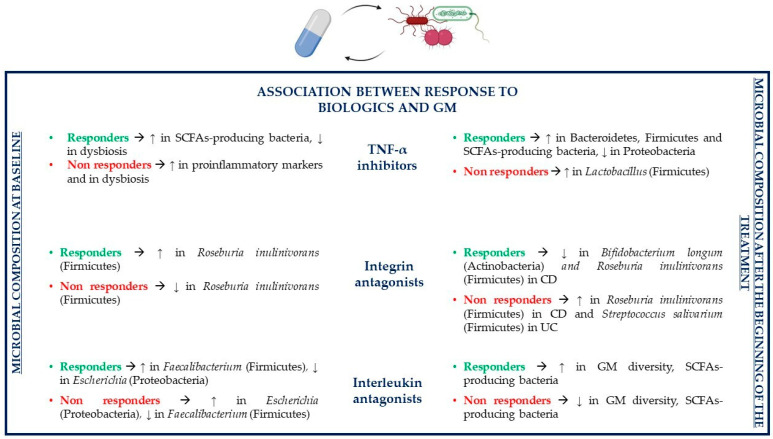
Association between the GM and biologic drugs (CD, Crohn’s disease; GM, gut microbiota; SCFAs, short-chain fatty acids; TNF-α, tumor necrosis factor α; UC, ulcerative colitis).

The GM profile before treatment has been associated with the response to anti TNF-α therapy. Before the start of TNF-α therapy, responders and non-responders to treatment have a different GM composition, AMP expression in both serum and sigmoid colonic mucosa and cytokine profile in both serum and sigmoid colonic mucosa [126,127]. More precisely, one study showed that at baseline responders have a higher expression of particular AMPs, such as defensin 5 and eosinophilic cationic protein and of proteins able to regulate AMP expression or having antimicrobial functions, such as bactericidal permeability-increasing protein (BPI), histone H1.5 (HIST1), 40S ribosomal protein S19 (RPS19), high-mobility group protein B1 (HMGB1) and others. Moreover, lower dysbiosis and increased levels of SCFAs and of *Faecalibacterium prausnitzii* (Firmicutes) have been reported (Figure 5). *Faecalibacterium prausnitzii* concentrations may increase also during TNF-α inhibitors treatment. On the contrary, non-responders show high levels of the cathelicidin AMP, increased levels of proinflammatory mediators such as IL-6, IL-12a, IL-17a and TNF-α and detectable expression of histone deacetylase 1, which can inhibit the expression of AMPs [127]. Furthermore, a second study showed that non-responders have increased levels of proinflammatory mediators such as IL-1β, IL-6, IL-17a, TNF-α and IFN-γ [126].

Various studies showed that anti TNF-α agents reduce inflammation by inhibiting the TNF-α cytokine but also by indirectly modulating the GM in order to restore a bacterial composition similar to that present in healthy individuals. Jones-Hall and colleagues showed that the GM of IBD patients treated with anti TNF-α drugs is characterized by a decreased abundance of *Enterobacteriaceae*, especially *Escherichia coli* (Proteobacteria)*,* and *Ruminococcus* (Firmicutes) and by an increased abundance of Bacteroidetes and Firmicutes phyla [128]. Other studies described that patients responding to TNF-α inhibitors have reduced levels of potentially pathogenic Proteobacteria and higher concentrations of beneficial SCFAs-producing bacteria in comparison to untreated individuals. Indeed, the levels of SCFAs-producing *Coprococcus* and *Roseburia inulinivorans* (Firmicutes) are reduced at the beginning of TNF-α treatment compared to healthy individuals, but their abundance and the abundance of SCFAs increases during treatment until it equals the levels of healthy subjects. The gut bacterial ecosystem is particularly disrupted in patients, which are not able to achieve remission with TNF-α treatment. An increase in the abundance of *Lactobacillus* (Firmicutes) is linked to non-remission. The exchange of metabolites between bacterial organisms, especially as regards butyrate and substrates involved in its synthesis, is more than 80% lower in non-remitting patients compared to patients achieving remission with TNF-α inhibitors. Indeed, a reduction in bacterial species involved in SCFAs production has been associated with TNF-α treatment failure [103,129]. 

IBD-related symptoms have an inversely proportional relationship with infliximab concentrations, but they have been also associated with infliximab-related GM modifications [130]. Indeed, Seong and colleagues reported that between the first and the seventh week after the first infliximab maintenance administration, gut dysbiosis improves and small changes in the GM composition can be observed, which make it more similar to that of healthy individuals compared to that of untreated patients; furthermore, an improvement in intestinal dysbiosis has been reported even during induction therapy with infliximab. The GM of both the first and the seventh week is dominated by Firmicutes and Bacteroidetes, followed by Actinobacteria and Proteobacteria [130]. Other studies showed that after infliximab treatment, the levels of *Actinomycetales* (Actinobacteria) decrease; instead, the levels of *Anaerostipes, Blautia, Coprococcus, Faecalibacterium, Lachnospira* and *Roseburia* (Firmicutes) increase; the latter are all SCFAs-producing bacteria, which have an important role in the response to treatment because of their immunomodulatory and anti-inflammatory effects [130,131]. Additionally, Dovrolis and colleagues reported that after infliximab treatment, CD patients have a reduction in the abundance of Fusobacteria and an increase in Proteobacteria and *Ruminococcus* (Firmicutes); *Eubacterium* (Firmicutes)*, Escherichia* and *Shigella* (Proteobacteria) proportions increment especially in responders. In UC patients treated with infliximab, there is an increase in Bacteroidetes, *Veillonella* and *Ruminococcus* (Firmicutes) in responders and an increment in Actinobacteria in the group of non-responders [132]. 

One of the problems of anti TNF-α agents such as infliximab is the loss of response to treatment. Low trough levels of infliximab and the presence of antidrug antibodies are two factors involved in loss of response; however, drug trough levels are thought to be a better predictor of either response or loss of response to treatment than antidrug antibodies [133]. It has been shown that patients in remission have higher trough levels of infliximab compared to those not in remission or those who relapse, which is associated with a higher chance of mucosal healing and a lower probability of loss of response to treatment. However, this is not always true, as many IBD patients suffer from relapses despite high trough levels or maintain remission despite low trough levels, which led to the assumption that drug levels alone are not enough to predict clinical outcomes [134]. Indeed, it has been noted that patients with higher trough levels of infliximab who achieve mucosal healing generally also have a richer GM compared to individuals with lower trough levels. Infliximab-associated mucosal healing leads to higher microbial diversity compared to the non-mucosal healing group by incrementing the abundance of *Bacteroides* (Bacteroidetes) and Firmicutes such as *Blautia* and *Faecalibacterium*, and by leading to a decrease in the levels of Bacteroidetes, especially *Prevotella*. Beneficial *Faecalibacterium prausnitzii* (Firmicutes) concentrations depend on both infliximab trough levels and mucosal healing, as they are increased in patients that present both of these conditions [130]. 

Additionally, adalimumab is able to change the GM composition in order to make it shift towards a GM similar to that of healthy individuals and to normalize dysbiosis [135]. During treatment of IBD patients with adalimumab, the predominant microbial phylum and genus are Firmicutes and *Clostridium* (Firmicutes), respectively [128]; on the contrary, Busquets and colleagues reported that levels of *Clostridium* decreased during IBD therapy. Other bacteria that increase during adalimumab therapy are Actinobacteria, Bacteroidetes, especially the *Bacteroides* genus, and *Faecalibacterium prausnitzii* (Firmicutes), but also other butyrate-producing bacteria increment in abundance [135]. Another study revealed that levels of Actinobacteria increase particularly in case of colon inflammation. In case of normalization of C reactive protein (CRP) and remission, *Lachnospiraceae* (Firmicutes) levels increase proportionally to the remission rate, thus leading to higher concentrations of SCFAs-producing bacteria. In patients responding to treatment *Bifidobacterium adolescentis* (Actinobacteria) and Proteobacteria levels are reduced [136]. More precisely, potentially pathogenic *Escherichia coli* (Proteobacteria) abundance greatly decreases alongside continuation of treatment and especially in responders. Some species of the *Ruminococcus* (Firmicutes) genera such as mucolytic *Ruminococcus gnavus*, whose increase in CD patients provokes mucosal damage, decrease after administration of adalimumab [135,136]. As for infliximab, also for adalimumab, there is an association between higher trough drug levels and higher rate of endoscopic remission (mucosal healing), which may be linked to adalimumab-related GM alterations too. Greater adalimumab trough levels and higher mucosal healing lead to the normalization of CRP and serum albumin levels by decreasing the former and increasing the latter; higher serum albumin concentrations are linked to a greater adalimumab therapeutic action because of a decreased drug clearance and an increased drug half-life. Furthermore, adalimumab treatment modulates GM composition based on endoscopic activity: the Bacteroidetes phylum is more represented in patients with mild-to-moderate endoscopic activity compared to those with severe activity; instead, the phylum Proteobacteria is more represented in patients with severe endoscopic activity [136,137,138,139]. 

As for infliximab and adalimumab, certolizumab pegol is also able to improve mucosal lesions in nearly half of IBD patients and reduce their severity by increasing endoscopic responses and remission rates; however, its relationship with the GM is still unknown [140]. As regards golimumab, a study has shown that some bacterial taxa differ in responders compared to the placebo group and patients in remission have a different GM from patients who are not able to achieve remission [141]. There is still very little information about golimumab’s effects on the GM; however, a clinical trial concerning the association between golimumab and the GM is being conducted [142] (Table 1).

#### 4.5.2. Integrin Receptor Antagonists

Integrin receptor antagonist biologic drugs are used to treat IBD patients who are incapable of achieving remission with conventional therapy. Indeed, these are antiadhesion antibodies that prevent leukocyte migration from the circulation to the inflamed areas of the gut by targeting specific integrins such as the α4-integrin (natalizumab), the α4β7-integrin dimer (vedolizumab) or the β7-unit of the α4β7-integrin (etrolizumab) [143,144]. These drugs block the binding between the α4-integrin family and some cellular adhesion molecules (CAMs), which avoids lymphocyte migration to the inflamed areas [145]. Because natalizumab is not a gut-selective anti-inflammatory agent leading to a reduced T-cell immunosurveillance of the central nervous system, a gut-selective integrin receptor antagonist vedolizumab has been developed [146,147]. Etrolizumab has a dual mechanism of action because, despite preventing leukocyte migrations, it is also able to avoid gut intraepithelial lymphocyte retention [148].

The GM can predict responses to IBD treatment with integrin receptor antagonists. Ananthakrishnan and colleagues showed that before starting treatment with vedolizumab, CD patients who will respond to the drug have higher levels of *Roseburia inulinivorans* (Firmicutes), which produces anti-inflammatory SCFAs and encodes some genes for flagellin proteins able to encourage proinflammatory IL-8 synthesis and of a *Burkholderiales* (Proteobacteria) species compared to non-responders; in UC, these differences are not statistically significant (Figure 5). Additionally, the pathways involved in branched chain amino acid (BCAA) biosynthesis are enriched in individuals who will be ustekinumab responders; BCAAs are able to reduce gut inflammation and mucosal damage, upregulate some AMPs, control cytokine production and decrease oxidative stress. At baseline, *Bifidobacterium longum* (Actinobacteria) and *Dialister invisus* (Firmicutes) contribute to the development of some strain specific single nucleotide polymorphisms (SNPs) localized in the pathways of L-arginine biosynthesis, which will be present only in CD responders. In UC, some bacterial SNPs involved in uridine monophosphatate biosynthesis pathway and pentose phosphate pathway can be identified in baseline samples of responders to vedolizumab treatment; these SNPs are observed mainly in *Bifidobacterium longum* (Actinobacteria)*, Ruminococcus torques* (Firmicutes) and *Escherichia coli* (Proteobacteria) [149]. Despite depending on the GM, clinical response to vedolizumab therapy depends also on other factors. More precisely, higher rates of clinical response and remission are associated with less severe baseline disease activity, lack of prior exposure to TNF-α inhibitors and subsequent loss of response, lower levels of inflammatory markers and higher baseline α4β7 or αEβ7 integrin expression in the gut mucosa [150,151].

GM contributes to the modulation of the treatment response to integrin receptor antagonists, but these drugs can affect the GM composition as well. Levels of bacteria such as *Bifidobacterium longum, Eggerthella* (Actinobacteria)*, Roseburia inulinivorans, Ruminococcus gnavus* and *Veillonella parvula* (Firmicutes) decrease in CD patients in remission in comparison to untreated individuals. On the contrary, *Roseburia inulinivorans* (Firmicutes) abundance is increased in CD non-responders; instead, in UC non-responders, *Streptococcus salivarium* (Firmicutes) concentrations increment. In CD, the nicotinamide adenine dinucleotide (NAD) salvage pathway is reduced in patients achieving remission, which leads to a decrease in oxidative stress; meanwhile, in UC, palmitate and stearate biosynthesis is increased in responders and reduced in non-responders [149].

To the authors’ knowledge, the only study considering the effects of vedolizumab on the GM is the one just mentioned, which was written by Ananthakrishnan and colleagues. The McMaster University is conducting a clinical trial on the effects of a combination therapy with vedolizumab and fecal microbiota transplant on fecal and mucosal microbiota in UC patients, both those in remission and those with active disease [152] (Table 1).

#### 4.5.3. Interleukin Receptor Antagonists

Proinflammatory cytokines such as interleukins IL-12 and IL-23 increase gut inflammation in IBD because they induce the differentiation of naïve T cells to Th1 and Th17, respectively, which consequently leads to the upregulation of proinflammatory cytokines and gut inflammation. More precisely, IL-12 interacts with receptors on T cells and NK cells to induce the maturation of naïve T cells to Th1 cells that than produce TNF-α and IFN-γ. Instead, IL-23 binds to its receptor, which activates Th17 cells that develop IL-6, IL-17, IL-21 and IL-22. Therefore, one of the treatment options for IBD patients failing TNF-α inhibitors’ therapy is the anti-p40 monoclonal antibody ustekinumab, a biologic drug that inhibits both IL-12 and IL-23 [143,153,154,155]. Another anti-p40 antibody blocking IL-12/IL-23 is briakinumab; instead, drugs such as brazikumab, mirikizumab and risankizumab are anti-p19 medications that selectively inhibit only IL-23; however, the latter have not yet been approved for the treatment of IBD, as they are still undergoing clinical trials to prove their efficacy and safety [156]. 

Baseline GM composition can predict therapeutic responses of IBD patients treated with the interleukin antagonist ustekinumab, so it can help in identifying patients who will be more likely to respond and achieve remission after ustekinumab treatment. More precisely, some studies have reported that *Bacteroides* (Bacteroidetes) and *Faecalibacterium* (Firmicutes) levels are increased in TNF-α refractory CD patients who will achieve remission after ustekinumab therapy in comparison to non-responders; instead, *Escherichia* and *Shigella* (Proteobacteria) are less abundant, suggesting that all these bacteria positively or negatively affect IBD pathogenesis (Figure 5). Lower baseline α-diversity (different microbial composition in a single ecosystem) is associated with longer disease duration, but there are no significant associations between α-diversity and inflammatory biomarkers such as CRP, fecal calprotectin or fecal lactoferrin, contrarily to β-diversity (variations of the microbial composition in different ecosystems), which is correlated to CRP, calprotectin and lactoferrin levels [157]. Apart from GM composition and diversity, other predictors of primary response to ustekinumab have been studied. However, it seems that patient-related factors and disease-related factors, duration and location of disease are not associated with response to treatment, so further studies are needed to individualize predictors of response to interleukin antagonists [150,158]. 

It is also possible that the therapy with ustekinumab could alter the GM. Indeed, the microbial diversity of patients in remission after treatment is higher compared to baseline diversity of those who will enter remission. On the contrary, the GM of non-responders and that of the placebo group are not significantly different from the baseline GM. The GM composition varies between patients in remission and those with active disease: *Blautia, Clostridium XIVa, Faecalibacterium, Roseburia* and *Ruminococcaceae* (Firmicutes) are all more abundant in remitters compared to patients with active disease, confirming that SCFAs-producing bacteria have a protective role in IBD [157]. 

The fact that the levels of *Clostridiales* such as *Roseburia* (Firmicutes) increase following treatment with TNF-α inhibitors or interleukin antagonists in responders but decrease in patients responding to integrin receptor antagonists may be explained by the fact that the GM could affect to a greater extent the response to those biologic drugs, which have a more systemic effect in comparison to those who are more specific. Indeed, vedolizumab has a specific effect on leukocyte trafficking; instead, both anti TNF-α agents and interleukin antagonists act on T-cell pathways [120] (Table 1).

## 5. Conclusions

IBD is linked to alterations in the composition, function and activity of the bacterial species of the GI tract. The intestinal barrier also has an important role in the pathogenesis of IBD. Indeed, in IBD patients, this barrier is compromised, thus leading to an increased permeability and an excessive bacterial translocation able to cause an aberrant stimulation of the immune system. 

There are also several studies that confirm the microbial ability to influence pharmacological activities of medications used in IBD treatment and the ability of the same drugs to impact on the GM composition. The GM can enhance drug actions and consequently increase therapeutic responses, or instead, it can decrease responses to treatment by inactivating drugs. The GM can also metabolize drugs to toxic metabolites and, thus, be responsible for some adverse effects. For instance, bacteria belonging to the GM are involved in the metabolism of various IBD drugs such as 5-ASA, antibiotics, corticosteroids, thiopurines and methotrexate. Therefore, the GM-mediated metabolism is either necessary for the activation and the consequent therapeutic effects of these drugs or is responsible for the production of inactive or toxic metabolites. More precisely, bacterial azoreductases release 5-ASA from its prodrugs; instead, some enzymes belonging to various bacterial species inhibit the pharmacological effects of the drug by acetylating it, and other enzymes may contribute to adverse effects by producing a toxic 5-ASA metabolite. Active corticosteroids can be released from their prodrugs or can undergo degradation reactions because of bacterial enzymes. Thiopurines can be either activated or inactivated by the GM: the activation is due to the ability of some bacterial strains to convert these drugs in their active metabolites, while the inactivation occurs through a methylation reaction depending on several microorganisms. Lastly, some bacterial species convert methotrexate polyglutamates to the mono-glutamate form and eventually to non-toxic metabolites. Additionally, the baseline GM composition has been associated with response to the consequent treatment with some IBD drugs such as anti TNF-α agents, integrin receptor antagonists or interleukin antagonists. Indeed, at baseline, the GM of responders to treatment is characterized by lower dysbiosis, lower levels of potentially pathogenic bacteria and higher levels of beneficial microorganisms compared to the GM of non-responders. 

On the contrary, drugs can affect the GM by incrementing or reducing its diversity and richness. In most cases, the drugs used to treat IBD increase the levels of some beneficial bacteria producers of anti-inflammatory SCFAs and decrease the levels of pathogenic microorganisms. However, it is not clear if these variations are a consequence of the direct effects of IBD drugs on gut bacteria or of the immunosuppressive effects of drugs on IBD symptoms.

Therefore, the GM–drug relationship can be either beneficial or disadvantageous. 

Currently, the evidence suggesting the role of the GM in the pharmacological responses to IBD treatment is still limited. Therefore, new studies are needed for a better understanding of the association between the GM and the drugs used for the treatment of IBD in order to determine if changes in the microbial baseline composition could be considered markers of treatment response and how the inter-individual differences in the GM will affect the actions of IBD drugs. The ultimate goal exposed by various scientists is to develop predictive models for disease or for pharmacological responses to prevent the development of pathogenic conditions and to establish a personalized drug treatment that will increase the chances for drug response, decrease failures and the risks for adverse reactions.

A routine evaluation of the GM-related drug metabolism during the development of drugs has been suggested in order to determine if the GM will regulate drug efficacy and/or toxicity. Firstly, the identification of bacteria having xenobiotic-metabolizing properties, especially as regards bacteria resident in the gut, and the identification of both GM-mediated reactions and of the drug metabolites produced from these reactions will be required. Additionally, the use of animals or alternative models to determine inter-individual differences in the GM–drug relationship and, thus, in the inter-individual variations of drug efficacy and/or toxicity should be considered. 

Furthermore, despite pharmacological treatment, there are multiple environmental factors such as ethnicity, lifestyle and smoking, which can influence the microbial richness and/or diversity. Because of the large number of environmental factors to consider and because of the difficulty in examining their influence on the GM, the studies of the relationship between the environment and the GM are quite complex.

## Figures and Tables

**Table 1:** Most relevant information about the association between the GM and drugs used for the treatment of IBD (5-ASA, 5-aminosalicylic acid; AMPs, antimicrobial peptides; APA, 4-amino-4-deoxy-N10-methylpteroic acid; AZA, azathioprine; CD, Crohn’s disease; DAMPA, 2,4-diamino-N-10-methylpteroic acid; MAP, mycobacterium avium subspecies paratuberculosis; MP, mercaptopurine; MTX, methotrexate; MTX-PGs, methotrexate polyglutamates; SCFAs, short-chain fatty acids; TGN, thioguanine nucleotides; TPMT, thiopurine S-methyl transferase; UC, ulcerative colitis).

**Drug**	**Modulation by Microbial Enzymes**	
**5-ASA**	Release of 5-ASA from prodrugs via azoreductases produced by *Clostridium, Eubacterium, Staphylococcus aureus, Escherichia coli, Klebsiella aerogenes, Pseudomonadaceae* and *Bacteroides* Acetylation of 5-ASA into an inactive compound via N-acetyltransferases produced by various Bacteroidetes, Firmicutes and Proteobacteria strains such as *Pseudomonas aeruginosa*	
**Corticosteroids**	Degradation into inactive compounds Release from prodrugs via hydrolysis Reduction	
**Thiopurines**	Conversion to active TGN via enzymes produced by *Escherichia coli, Enterococcus faecalis, Bacteroides fragilis, thetaiotaomicron* and *vulgatus* Methylation to methylated thiopurine metabolites via an orthologue of human TPMT produced by *Pseudomonas aeruginosa, fluorescens, ovalis* and *syringae* Conversion to inactive compounds via xanthine oxidase produced by *Escherichia coli*	
**Methotrexate**	Hydrolysis into non-toxic and less effective APA Conversion to active MTX-PGs Reconversion to MTX from MTX-PGs via deamidation enzymes produced by some *Pseudomonas* strains Conversion to non-toxic DAMPA via glutamate carboxypeptidase II produced by *Pseudomonas*, *Prevotellaceae* and *Anaeroplasmataceae* or via the orthologue enzyme produced by *Escherichia coli*	
**Drug**	**Microbial Composition at Baseline**	**Microbial Composition after Beginning of Treatment**
**TNF-α inhibitors**	Responders → ↑ in SCFAs-producing bacteria and in the expression of some AMPs, ↓ in dysbiosis Non responders → ↑ in proinflammatory markers, in the expression of other AMPs and in dysbiosis	Responders → ↑ in Bacteroidetes, Firmicutes and SCFAs-producing bacteria, ↓ in Proteobacteria such as *Enterobacteriaceae* (*Escherichia coli*) and *Ruminococcus* Non responders → ↑ in Lactobacillus, ↓ in the exchange of metabolites such as butyrate between bacterial organisms
**Integrin antagonists**	Responders → ↑ in *Roseburia inulinivorans* and some *Burkholderiales* strains Non responders → ↓ in *Roseburia inulinivorans* and some *Burkholderiales* strains	Responders → ↓ in *Bifidobacterium longum, Eggerthella*, *Roseburia inulinivorans*, *Ruminococcus gnavus* and *Veillonella parvula* in CD Non responders → ↑ in *Roseburia inulinivorans* in CD and *Streptococcus salivarium* in UC
**Interleukin antagonists**	Responders → ↑ in *Bacteroides* and *Faecalibacterium*, ↓ in *Escherichia* and *Shigella* Non responders → ↑ in *Escherichia* and *Shigella*, ↓ in *Bacteroides* and *Faecalibacterium*	Responders → ↑ in GM diversity, *Blautia*, *Clostridium* XIVa, *Faecalibacterium*, *Roseburia* and *Ruminococcaceae* Non responders → ↓ in GM diversity, *Blautia*, *Clostridium* XIVa, *Faecalibacterium*, *Roseburia* and *Ruminococcaceae*
